# Antiurolithiasis Activity of Bioactivity Guided Fraction of* Bergenia ligulata* against Ethylene Glycol Induced Renal Calculi in Rat

**DOI:** 10.1155/2017/1969525

**Published:** 2017-03-02

**Authors:** Ikshit Sharma, Washim Khan, Rabea Parveen, Md. Javed Alam, Iftekhar Ahmad, Mohd Hafizur Rehman Ansari, Sayeed Ahmad

**Affiliations:** Bioactive Natural Product Laboratory, Department of Pharmacognosy and Phytochemistry, Faculty of Pharmacy, Jamia Hamdard, New Delhi 110062, India

## Abstract

Dried rhizome of* Bergenia ligulata* (pashanbhed) is commonly used as a traditional herbal medicine with a wide range of therapeutic applications including urolithiasis. Aqueous extract of* B. ligulata* was prepared through maceration followed by decoction (mother extract, 35.9% w/w). Further, polarity based fractions were prepared successively from mother extract which yielded 3.4, 2.9, 5.4, 7.5, and 11.3% w/w of hexane, toluene, dichloromethane (DCM), *n*-butanol, and water fractions, respectively. The in vitro, ex vivo, and real-time antiurolithiasis activity of mother extract and fractions were carried out using aggregation assay in synthetic urine and in rat plasma. The study revealed that DCM fraction has significantly (*p* < 0.05) greater inhibitory potential than other fractions. Ethylene glycol in drinking water (0.75%, v/v) for 28 days was used for induction of urolithiasis and the curative effects of mother extract and DCM fraction were checked for the level of oxalate, calcium, creatinine, uric acid, and urea of both urine and serum. Treatment with mother extract and DCM fraction at a dose of 185 mg/kg and 7 mg/kg, respectively, in ethylene glycol induced rats resulted in a significant (*p* < 0.05) decrease in serum and urine markers. Histological study revealed lower number of calcium oxalate deposits with minimum damage in the kidneys of mother extract and DCM fraction treated rats. This result provides a scientific basis for its traditional claims.

## 1. Introduction

Urolithiasis is the presence of calculi in the kidney and/or in any part of the urinary tract, including the ureters and bladder. Nearly about 80% of these calculi are composed of calcium oxalate and phosphate [1]. The recurrence rate of urolithiasis without any precaution or preventive treatment is approximately 10% per year [2]. Epidemiological studies revealed that the urolithiasis is more prone to men (12%) than women (6%) and is more prevailing with increasing ages between 20 and 40 in both men and women [3]. Urolithiasis is a multifaceted process which includes crystal nucleation, aggregation, and growth of insoluble particles [4]. It is assumed that when the urine becomes saturated with insoluble materials as a result of the excessive rate of excretions which leads to the formation of crystals and aggregates to form a stone [5], urolithiasis needs both preventive and curative therapy because of having a higher rate of reoccurrences of kidney stone.

At present, no suitable drugs in modern medicine are available for the management of urolithiasis. Several therapies which include thiazide diuretic and alkali citrate are used for preventive therapy but no allopathic medicine available which can dissolve the stone. The other types such as surgical removal of stones, extracorporeal shock wave lithotripsy (ESWL), and percutaneous nephrolithotomy (PCNL) are being used for the management of stones. Moreover, these are less convincing and cause side effects such as hemorrhage, hypertension, tubular necrosis, and subsequently fibrosis of the kidney [6].

Medicine from the plant is the alternative and last source for new drug and it remains the last choice to the physician for better relief. A number of plants have been used because of being efficient for curing and correcting urinary stones; for example,* Tribulus terrestris* (fruit),* Didymocarpus pedicellata* (leaf),* Dolichos biflorus* (seed), fruits and leaves of* Solanum nigrum*, and seeds of* Cichorium intybus* are very common in Traditional Indian System of Medicine [7]. These plants and their products are also reported to be effective in the treatment as well as prevention of recurrence of renal calculi with minimal or no side effects [8].

In Asia, mainly in India and Pakistan,* Bergenia ligulata* (family: Saxifragaceae) has been used for centuries for a wide range of diseases. But most importantly it has been used as antidiuretic and antilithotriptic agent.* B. ligulata* is used for the treatment of dysuria and strangury and for stones in the kidney and ureter. Some preliminary studies have evaluated the antiurolithic potential of* B. ligulata* rhizome. The aqueous extract of* B. ligulata* rhizome inhibited homogenous precipitation of CaC_2_O_4_ crystals [9] and in vitro growth of CaC_2_O_4_ and calcium hydrogen phosphate dihydrate crystals. The alcoholic extract of* B. ligulata* has been found effective in dissolving the calculi developed in the bladder of rats by foreign body insertion and reduced idiopathic hyperoxaluria in stone formers [10]. Only one study has been reported till date for a molecular-based mechanism with the support of antiurolithiasis effect of hydroalcoholic extract of its rhizome against ethylene glycol induced urolithiasis [10].

All of the studies used high dose (>200 mg/kg) for antiurolithiasis effect. But in our study we isolated bioactivity guided fractions from an aqueous extract of* B. ligulata* and further used it for the management of urolithiasis with a low dose. Most of the previous studies were performed on its preventive effect but we investigated the curative properties of the mother as well bioactivity guided fraction of* B. ligulata* against ethylene glycol induced calculi. Our study validated the previous report by providing some scientific mechanism using in vitro, in vivo, and ex vivo models. Apart from scientific validation, a complete quality controlled evaluation has been carried out for each bioactivity guided fraction as well as aqueous extract.

## 2. Materials and Methodology

### 2.1. Preparation of Plant Extract and Its Bioactivity Guided Fractionations

Dried and powdered rhizome of* B. ligulata* (500 g) was extracted by boiling with demineralized water (1 : 16, w/v) till water reduced to its 8th time of volume. The extract was filtered and the filtrate was evaporated on a boiling water bath until constant weight was obtained (mother extract). The aqueous extract thus obtained was suspended in double distilled water (1.0 g/10 mL) and sonicated for 15 min at 45°C. Polarity based fractionation of prepared aqueous suspension was done using equal proportions of hexane, toluene, DCM, and *n*-butanol (thrice each). The aqueous suspension left after fractionation was evaporated to dryness. The extractive values and % yields of different fractions were calculated and stored at 4°C for bioactivity and quality control analysis.

### 2.2. Quantitative Estimation of Catechin, Gallic Acid, Tannic Acid, and Quercetin in Plant Extract/Fractions

Mother extract and each fraction were prepared at the desired concentration in HPLC grade methanol separately. Further, it was filtered through a 0.45 *μ*M PTFE membrane filter and used for HPLC analysis. Filtered samples were stored at −20°C until analysis. Two different concentrations of each extract (5.0 or 10 mg/mL) were used for HPLC analysis and each sample was analyzed in triplicate and the mean concentration of each metabolite found was calculated from linearity plot. Separation and analysis of catechin, gallic acid, tannic acid, and quercetin were carried out through the newly developed and validated HPLC method [11]. Alliance HPLC system (e2695 Separation module, Waters, USA) integrated with a gradient pump attached with variable wavelength programmable photodiode array detector was employed for analysis. Chromatographic analysis was performed on Hypersil 5 *μ* C18 (ODS) column (150 × 4.6 mm, Phenomenex, USA) using mobile phase composed of 0.5% v/v formic acid in water (A) and acetonitrile (B) at a flow rate of 1.0 mL/min in gradient elution program (Initially 100% A, 0–5 min 90% A, 5–10 min 70% A, 10–15 min 30% A, 15–20 min 2% A, and 20–22 min 100% A) and the wavelengths were set at 254 nm (for quercetin) and 278 nm (for gallic acid, tannic acid, and catechin) with a total of 25 min run time.

### 2.3. Antiurolithiasis Activity of* B. ligulata* Extracts and Its Fractions

#### 2.3.1. In Vitro Aggregation Assay

Inhibition assay of oxalate crystals aggregation was carried out for mother extract and fractions using the protocol described by Atmani and Khan (2000) [12] with slight modification. Various concentrations of calcium chloride (CaCl_2_) and sodium oxalate (Na_2_C_2_O_4_) ranging from 1 to 40 mmol/L were examined in order to standardize their concentrations through crystallization assay. The final concentrations of calcium chloride and sodium oxalate were selected based on their sensitivity in spectrophotometer and turbidity of the solution. Solutions of calcium chloride and sodium oxalate were prepared at the final concentrations of 6.0 mmol/L and 6.5 mmol/L, respectively, in a buffer containing Tris 0.05 mol/L and NaCl 0.15 mol/L at pH 6.5. The calcium chloride solution (950 *μ*L) was mixed with 100 *μ*L of herb extracts at the different concentrations (100–1000 *μ*g/mL). Crystallization was started by adding 950 *μ*L of sodium oxalate solution. In the case of control experiment, 100 *μ*L of buffer was added to calcium chloride solution. The temperature was maintained at 37°C throughout for 1 h of the incubation period. The optical density (OD) of the crystallized suspension was monitored at 620 nm. The percentage of aggregation inhibition was then calculated by comparing the turbidity in the presence of extract with that obtained in the control using following formula [13]:

(1) Percentage  of  inhibition=1−TurbiditysampleTurbiditycontrol×100.

The growth of crystals was expected due to the following reaction:

(2) $$\begin{array}{c} {CaCl}_{2}+{Na}_{2}C_{2}O_{4}⟶{CaC}_{2}O_{4}+2NaCl \end{array}$$

#### 2.3.2. Ex Vivo Turbidity Assay

The oxalate crystal inhibition potential of* B. ligulata* extract and fractions was also carried out in rat plasma to provide the biological environment. The plasma sample was diluted with equal volume of calcium chloride and sodium oxalate (12 mmol/L each), separately. Resulting plasma solutions containing either calcium chloride or sodium oxalate were used for nucleation assay. The 950 *μ*L of plasma containing sodium oxalate (6.0 mmol/L) was mixed with 100 *μ*L of* B. ligulata* extracts/fractions at the different concentrations (100–1000 *μ*g/mL). Crystallization was carried out by adding 950 *μ*L of plasma containing calcium chloride (6.0 mmol/L). The mixture solution was incubated for 1 h and the temperature was maintained at 37°C. The OD of the crystallized suspension was measured at 620 nm and the inhibition potential was estimated by comparing with control. The percentage of aggregation inhibition was then calculated by comparing the turbidity in the presence of the extract with that obtained in the control using above formula [13].

#### 2.3.3. Real-Time Nucleation Assay in Confocal Microscope

All reaction solutions were preheated to 37 ± 0.2°C. Glass-bottomed Petri dishes were preheated and assembled on the stage of a confocal microscope (Leica Microsystem, GmbH; Model-ERG0PLATTE DMI). Oxalate solutions were added to the dishes followed by buffer (for control)/mother extract or DCM fraction (as a test sample) and calcium solutions. To obtain high contrast image, equimolar concentrations of calcium and oxalate of 1.0 mM were mixed. The total reaction volume of solution was 1.0 mL. Crystallization was allowed to proceed for 30 min. All precipitates were scanned and imaged using a helium/neon-laser (wavelength: *λ* = 632.8 nm), a 63x plane achromat oil immersion objective, a 90/10 mirror, and the LSM AF lite software version 1.0.0 (Leica Microsystem, GmbH). Before every experiment, a prealignment of the microscope was carried out and the focus adjusted to the interface between glass and reaction solution. After adding the calcium chloride (Ca^2+^), nucleating and growing crystals were imaged every 20 s for 30 min. To maintain the focal plane at the crystal glass interface, fine tuning of the focus was conducted during imaging. Images captured using helium/neon-laser represents surfaces of crystals generated at the crystal glass interface. Shape and size of generated crystals were evaluated and for growth kinetics three image sequences of replicate samples were taken by measuring through a ruler [14].

### 2.4. In Vivo Evaluation

#### 2.4.1. Animals

Healthy Wistar female rats weighing 150–200 g of equivalent age groups were obtained from central animal house facility of Jamia Hamdard. Prior to initiation of the experiment, all the protocols were reviewed and approved by the Institutional Animal Ethics Committee (Registration Number 173/GO/RE/S/2000/CPCSEA and Approval Number 1100) following guidelines of the Committee for the Purpose of Control and Supervision of Experiments on Animal (CPCSEA), Government of India. All the rats were randomly housed under hygienic conditions in polypropylene cages and allowed to acclimatize for one week. The animal facility was maintained at an ambient temperature of 25 ± 2°C with proper ventilation having 50–60% relative humidity with 12 h light and dark cycle. The animals were acclimatized to the laboratory conditions for one week prior to the experiments and provided with standard diet (JS Exports, Kanpur India) and water ad libitum.

#### 2.4.2. Ethylene Glycol Induced Urolithiasis

The antiurolithiasis activity of mother extract and the best bioactive fraction of* B. ligulata* were evaluated in ethylene glycol induced urolithiasis model in albino Wistar rats [15]. Animals were divided into six different groups, each containing six animals. The normal control group (Group 1) maintained a standard laboratory diet with drinking water ad libitum and was treated with vehicle (1% CMC) for 28 days. Except for sham control group (Group 2), all other remaining groups received calculi inducing treatment for 28 days, comprised of 0.75% v/v ethylene glycol in drinking water. After 28 days of ethylene glycol treatment, the urolithiasis control group (Group 3) received vehicle (1% sodium CMC) by oral gavage once daily for 21 days. Group 4 received mother extract of* B. ligulata* with a dose of 185 mg/kg by oral gavage once daily for 21 days and group 5 received DCM fraction with a dose of 7 mg/kg. While the positive control group received Neeri tablet with a dose of 70 mg/kg by oral gavage once daily for 21 days.

#### 2.4.3. Collection and Analysis of Urine

After 21 days of a treatment period, all rats were kept in individual metabolic cages with free access to drinking water, and urine samples of 24 h were collected. A drop of concentrated hydrochloric acid was added to the collected urine and stored at 4°C. After urine collection, total urinary excretion of calcium, phosphate, oxalate, uric acid, and albumin was measured by various biochemical kits (Span Diagnostics Ltd., Surat, India) according to manufacturer's instruction.

#### 2.4.4. Serum Analysis

After urine collection on 22nd day blood was collected retroorbitally from each rat under mild anesthetic condition and animals were sacrificed by cervical decapitation. Serum was separated by centrifugation at 10,000 rpm for 5 min and stored at −20°C, until analysis. Various serum parameters such as calcium, oxalate, phosphate, uric acid, urea, albumin, total protein, and creatinine were analyzed by using various biochemical kits (Span Diagnostics Ltd., Surat, India) according to the manufacturer's instruction.

#### 2.4.5. Preparation of Kidney Homogenate and Biochemical Estimation

The abdomen of the animal was cut to remove both kidneys of selected animal from each group. Isolated kidneys were cleaned with ice-cold phosphate buffer saline. A fixed weight of kidney from each animal was heated separately in 10 mL of 1 N hydrochloric acid for 30 min and homogenized. The homogenate was centrifuged at 5000 rpm for 10 min, and the supernatant was separated. The supernatant was subjected to analysis for calcium, oxalate, uric acid, and phosphate by using various biochemical kits (Span Diagnostics Ltd., Surat, India) as per manufacturer's protocol.

#### 2.4.6. Histopathological Study of Kidney, Liver, and Spleen

Kidney, liver, and spleen of animals were dissected. Isolated organs were fixed in 10% neutral buffered formalin, processed in a series of graded alcohol and xylene, and finally embedded in paraffin wax. Histological sections (about 5 *μ*m thickness) were prepared by microtomy and stained with hematoxylin-eosin (H&E) dye for histological examination. Histological slides were examined under a light microscope at 10x magnification. The histological changes such as hemorrhages, congestion, focal tubular swelling, vacuolar changes in the cytoplasm, changes in tubular epithelium, and interstitial fibrosis were observed to check changes in the kidney, while in the liver inflammation in lymphocyte, sinusoid and liver necrosis, and changes in Kupffer cell and central vein were investigated. Amyloid deposition, neutrophil, and macrophages in spleen were investigated.

### 2.5. Statistical Analysis

Values were expressed as the mean ± standard deviation (SD). Two-way analysis of variance (ANOVA) followed by “Bonferroni posttests” (Graph Pad, San Diego, CA) was used for statistical analysis. All the treatment groups were compared with the toxic control group. *p* values < 0.05 were considered statistically significant.

## 3. Results and Discussion

### 3.1. Yield of Mother Extract and Fraction

Deionized water was used for the extraction of plant materials through maceration. The maceration extraction was selected for study due to its high yields and called mother extract (35.9% w/w). The mother extract was further fractionated using hexane (3.4% w/w), toluene (2.9% w/w), DCM (5.4% w/w), *n*-butanol (7.5% w/w), and water (11.34% w/w). A total of 5.3% of mother extract was lost during polarity based fractionation. The yields of mother extract and its polarity based fractions were shown in Table 1.

### 3.2. Quantitative Analysis of Catechin, Gallic Acid, Quercetin, and Tannic Acid

**Figure 1:**
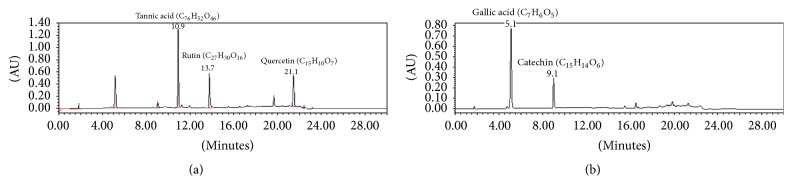
HPLC chromatogram of standard marker compounds at (a) 254 nm (tannic acid and quercetin) and at (b) 278 nm for gallic acid and catechin.

**Figure 2:**
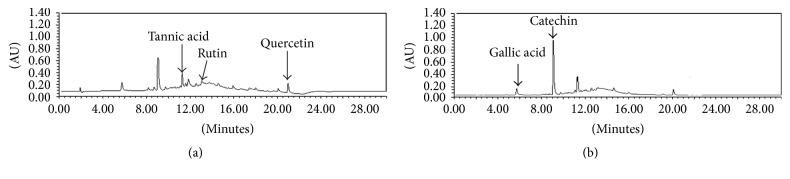
HPLC chromatogram of extracts at (a) 254 and (b) 278 nm indicating the presence of marker metabolites (tannic acid, quercetin, gallic acid, and catechin).

Analysis of catechin, gallic acid, quercetin, and tannic acid was evaluated on developed and validated simultaneous HPLC as shown in Figures 1 and 2. The contents of catechin, gallic acid, quercetin, and tannic acid are shown in Table 1. Mother extract had a maximum percentage of quercetin (4.2%), followed by tannic acid, gallic acid, and catechin. This quantitative analysis can be used for quality control analysis as well as for mechanism based evaluation of its therapeutic property. However, extract having more than 1.5% of quercetin can be used as a potent antiurolithiasis agent [16]. Among these markers, tannic acid was found as the most abundant marker in best bioactive (DCM) fraction. Catechin was found in both mother extract and DCM fraction, for which it can protect against oxidative injury by oxalate and crystal deposition along with its protective effect against calculi formation [17].

### 3.3. Antiurolithiatic Potential of Extract in Synthetic Urine

**Figure 3:**
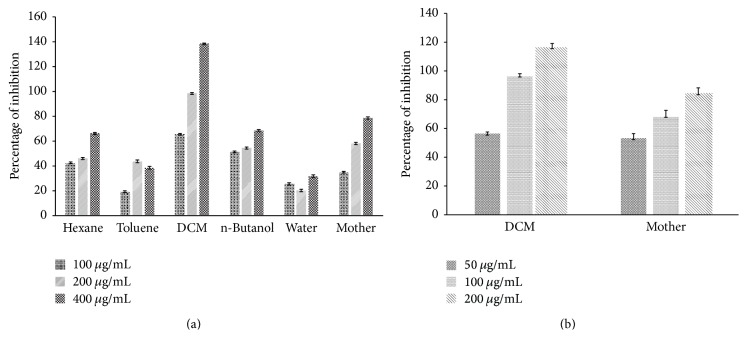
Effect of different concentrations of extract and fractions of* B. ligulata* on CaO_*x*_ crystallization in synthetic urine (a) and in plasma (b).

Calcium oxalate and calcium phosphate are the two major type crystals found in kidney stones. Over the period of time, crystals combine to form a small, hard mass called stones. Further, calcium oxalate stones have been classified into two types, that is, calcium oxalate monohydrate stones (COM) and calcium oxalate dihydrate (COD) [18]. DCM fraction and mother extract of* B. ligulata* had greater capability to inhibit the aggregation of calcium oxalate as a foremost element for stone forming in the urinary tract as shown in Figure 3, that is, the graph of percentage of inhibition of the crystallization of calcium oxalate with different concentrations of the aqueous extract and all its fractions of* B. ligulata*.

### 3.4. Effect of Extracts and Fractions on Real-Time Nucleation Assay

**Figure 4:**
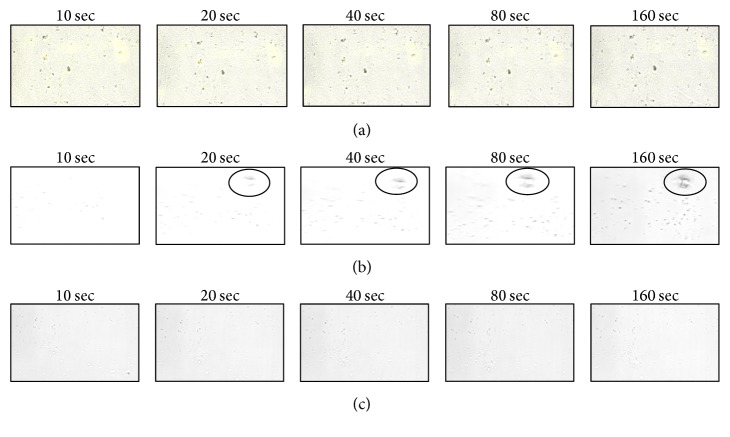
SCIM image sequences of calcium oxalate precipitate show no increase in crystal size in (a) mother extract and in (b) DCM fraction treated but in (c) control reaction solution crystal shape was increased with respect to time.

The first calcium oxalate crystal was observed after 5 min of incubation and then throughout the reaction time; the observed crystals were found to be precipitating. For analysis of precipitating crystals, measurements started when a microscopic field having a calcium oxalate crystal was encountered. Crystals as small as 3.0 *μ*m could be detected in microscope due to the high resolution of the imaging method.  Figure 4 shows an image sequence of growing crystals under control (Figure 4(a)) and drug supplemented (Figures 4(a) and 4(c)) condition. In the control condition, the size of the crystals was gradually increased (3.4–10.8 *μ*m) whereas, in drug supplemented reaction, no increase in the size of crystals was observed. Initially whatever calcium oxalate crystals were formed and further no increment in size of stone was observed in the presence of extract/fractions, concluding that both extract and fractions have preventive action on stone formation rather than curative properties to break down the calcium oxalate crystal. This study shows that scanning of calcium oxalate crystals under the confocal microscope is a powerful technique for determining the mode of action of any drug which inhibits nucleation and aggregation for calcium oxalate crystals precipitation.

### 3.5. Ex Vivo Antiurolithiasis Potential of Plant Extracts

In order to provide the biological environment, the antiurolithiasis activity of an extract of* B. ligulata* was carried out in rat plasma. DCM fraction and mother extract were examined in ex vivo assay. It was found that both extracts were responsible for the inhibition of calcium oxalate crystals formation. Mother extracts showed 68.11% inhibition after 30 min of incubation whereas DCM fraction showed 96.32% inhibition. In ex vivo conditions, better results were observed than in vitro. This phenomenon may be described as inhibition of calcium oxalate crystals which is due to inhibition of glycolate oxidase (GOX) by quercetin [19]. In our analysis, a significant amount of quercetin was found in mother extract and DCM fraction. This ex vivo protocol can be used for analysis of the antiurolithiasis potential of* B. ligulata* extract in other biological samples also.

### 3.6. Microscopic Examination of Urine

**Figure 5:**
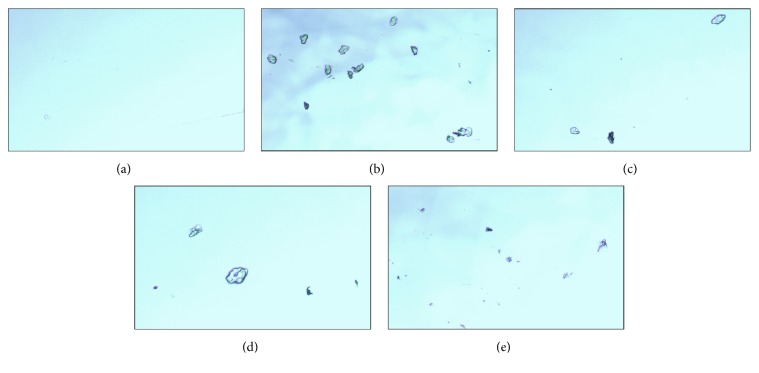
Calcium oxalate crystals, viewed under a microscope at 10x magnification in the urine of (a) normal control, (b) urolithiasis control, (c) DCM fraction treated, (d) mother extract, and (e) Neeri treated rat.

The microscopic evaluation of collected urine was done at 10x magnification. No crystals were found in the urine of normal control group but large peritubular crystals of CaO_*x*_ with the characteristic of rectangular shape scattered throughout were found in the urine of urolithiasis rats (Figure 5(b)). The microscopic evaluation revealed that the urine of mother extract, DCM fraction, and Neeri treated group rats was having lower number of crystals as compared to urolithiasis rat (Figure 5). DCM fraction and Neeri treated group showed few or almost dissolved small crystals. This microscopical evaluation of urine provides an evidence of curative properties* B. ligulata* on CaO_*x*_ calculi.

### 3.7. Evaluation of Physiological Parameters in Blood and in Urine

Effects of mother extract and DCM fraction of* B. ligulata* on urine and blood parameters of Wistar rats are shown in Tables 2 and 3. Parameters like calcium, urea, creatinine, oxalate, and uric acid in urine and serum were remarkably increased in urolithiasis control group. On treatment with mother extract 185 mg/kg of BW and DCM fraction 7 mg/kg of BW, the increased levels of these parameters were significantly decreased. But the level of decrease in these parameters was less in mother extract treated group as compared to DCM fraction treated rat. No significant changes have been observed in total protein of urolithiasis control group as compared with normal control rat, but the level of albumin was significantly increased in urolithiasis rat as compared to normal control rat and elevated level of albumin was reduced in both the experimental groups as compared to urolithiasis rat. Various important serum markers for kidney stone test such as calcium, oxalate, phosphate, uric acid, urea, and creatinine were increased in urolithiasis rat as compared to normal control rats whereas these elevated levels were ameliorated in mother extract as well as in DCM fraction treated group. No significant changes were observed in the sham control group as compared to normal control rat, indicating no adverse effect.

### 3.8. Effect of Mother Extract and DCM Fraction of* B. ligulata* on Kidney Homogenate Parameters

The deposition of urolithiasis promoters in kidney such as calcium, phosphate, oxalate, and uric acid was recorded. However, these promoters were found to be significantly (*p* < 0.05) higher in kidney homogenate of ethylene glycol alone treated urolithiasis control rats compared to the normal control rats. Treatment with mother extract and DCM fraction of* B. ligulata* in ethylene glycol induced urolithiasis produced a significant (*p* < 0.01) reversal in the deposition of urolithiasis promoters compared with urolithiasis control rats (Table 4).

### 3.9. Histopathological Evaluation of Kidney, Liver, and Spleen

**Figure 6:**
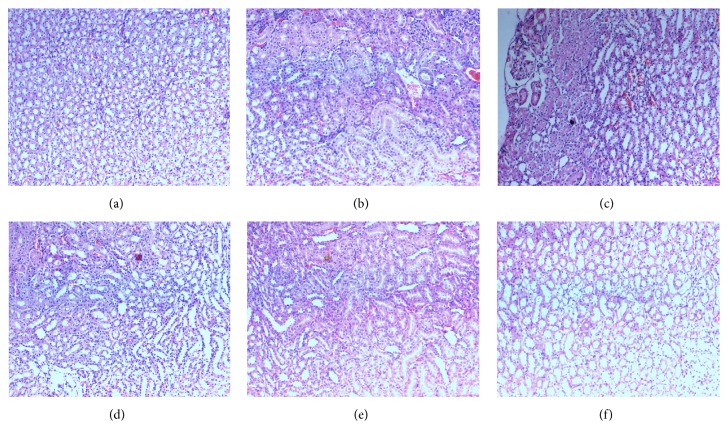
Microscopic images of kidney sections under a light microscope (40x) after hematoxylin and eosin staining from animals of (a) normal control, (b) sham control, (c) urolithiasis control, (d) mother extract, (e) DCM fraction, and (f) Neeri tablet treated rats.

The histopathological evaluation of the kidney of experimental animals was represented in Figure 6. Histopathological section of normal control and sham control animal's kidney revealed no abnormalities like interstitial inflammation and proximal tubules dilation within the renal tissue (Figures 6(a) and 6(b)). Kidney of urolithiasis group's animal showed marked inflammation in the interstice of the renal tissue along with proximal tubules dilation and deposition of the intratubular and interstitial crystal inside the tubules was a found as a characteristic sign of calculi development on continuing administration of 0.75% ethylene glycol (v/v) (Figure 6(c)). But the numbers of intratubular and interstitial crystals inside the tubules of both mother extract and DCM fraction treated rats (Figures 6(d) and 6(e)) and Neeri treated rats (Figure 6(f)) were reduced in comparison to urolithiasis group. The histopathological examination of the kidney sections has supported the findings from serum and urine parameters. Mother extract and DCM fraction treatment resulted in a reduction of the degenerative changes in the kidney tissue like interstitial infiltration of the inflammatory cell, epithelial cell dissociation, and proximal tubules dilation (Figure 6(d)).

A sectional view of the liver of normal control and the sham control group showed no inflammation, no dilation, and no congestion in vein and no amyloid deposition and no proliferation in Kupffer cells (Supplementary Figures 1(a) and 1(b) in Supplementary Material available online at https://doi.org/10.1155/2017/1969525). However, less inflammation and less congestion in central vein have been observed in mother extract as well as DCM fraction treated rat's liver as compared to urolithiasis rat (Supplementary Figures 1(b), 1(c), and 1(d)). The above result suggested that with the mentioned dose there was no effect on the liver. Similarly, no changes have been observed in the spleen of sham and normal control rat. But a little amyloid deposition in red pulp and around white pulp was observed in both urolithiasis and drug treated groups but it was lesser in extract treated rat as compared to urolithiasis rat.

Results revealed that no marked damage in the hepatic structure and spleen was observed by using given concentration of mother extract and DCM fraction (Supplementary Figure 2). Rather than damaging the hepatic structure, it has a protective effect on liver spleen and it may be due to the presence of high amount of quercetin [20]. The dose is also a major concern for toxicity of plant extract, higher dose leading to toxicity of liver and spleen, but histopathological results supported the given dose for its therapeutic efficacy without any toxic effect.

## 4. Discussion

The addition of sodium oxalate solution on calcium chloride solution resulted in the formation of calcium oxalate crystals. The effect of mother extract and DCM fraction of* B. ligulata* on CaO_*x*_ crystallization kinetics was investigated by the time course measurement of size and appearance of crystals. At the beginning of experiment some small size crystals were formed and further no increment in size and number of the crystals was observed in DCM fraction and mother extract treated experiment (Figure 4). However, the experiment showed continuous increment of crystal size and number in absence of extract. In this study, both mother extract and DCM fraction inhibited the CaO_*x*_ crystal aggregation in a concentration-dependent manner. In the incubation study, mother extract and DCM fraction of* B. ligulata* caused a decrease in crystal turbidity which is in agreement with Guerra et al. (2004) [21]. A critical step in the development of clinically symptomatic stone from a free particle is crystal retention. However, crystal agglomeration has long been identified as an important process leading to crystal retentions. In urine, physiological urolithiasis inhibitor like citrate was found which decreased the saturation of CaO_*x*_ and inhibited crystal nucleation, growth, and aggregation. To prevent the recurrence of stone disease, a possible therapeutic strategy may be the interference with crystal growth and aggregation. The hydroalcoholic extract of* B. ligulata* rhizome is previously reported to inhibit CaO_*x*_ crystal precipitation and growth [10]. Our experimental results support the previous studies and also ascertain the presence of CaO_*x*_ crystal aggregation inhibiting constituents in* B. ligulata*.

In the present experiment, we demonstrated the protective antiurolithiasis properties of both mother extract and DCM fraction of* B. ligulata* in experimentally induced Wistar rat. In our study, the mother extract was subjected to polarity based fractionation using hexane, toluene, DCM, and n-butanol. Further, the best fraction was selected on the basis of in vitro antiurolithiasis activity. DCM fraction was selected as the best fraction and it was further deployed for ex vivo analysis. The inhibitory potency of the plant was tested on the nucleation and growth of the most commonly occurring kidney stones, calcium oxalate monohydrate. A concentration-dependent trend of inhibition was observed using mother extract of* B. ligulata* and its DCM fraction with maximum inhibition of CaO_*x*_ growth as compared to other fractions. Our reports are in agreement with the studies previously reported on the antiurolithiasis potency of* B. ligulata* on the growth of CaO_*x*_ crystals using double diffusion gel growth technique [10]. The ex vivo study represents one of the first uses of plasma rather than using synthetic urine to check the antiurolithiasis potential of any herbal formulation. In our ex vivo finding, a significantly better result has been observed as compared to synthetic urine. This ex vivo study can be used as a model to check the antiurolithiasis potential of extracts/medicine. Some drug may give results in vitro but it may not work in in vivo model. While performing ex vivo activity, the results are usually reproducible in in vivo models. This study may work as a model to elaborate the mechanism behind its activity under a controlled environment.

Real-time assay of antiurolithiasis activity was carried out to evaluate its mechanism. We also examined the curative properties of mother extract and DCM fraction in ethylene glycol induced renal calculi in Wistar rat. Urinary supersaturation of various stone-forming elements is commonly considered to be one of the causative factors in the stone formation [3]. Ethylene glycol induced renal calcium oxalate (CaO_*x*_) crystal deposition is the most apposite animal model that is regularly used to mimic the stone formation in humans. Administration of ethylene glycol (0.75% v/v) with drinking water for 28 days significantly caused renal stone which is mainly composed of CaO_*x*_ [18] without producing any carcinogenicity or gastric ulcer [22]. To our knowledge, this is the first study on curative effect of mother extract and DCM fraction of* B. ligulata* in ethylene glycol induced urolithiasis. In our present study, ethylene glycol treatment significantly increased the level of oxalate in the urine of urolithiasis rats that was ameliorated by 21 days of treatment of both mother extract and DCM fraction. The decrease in oxalate excretion by both the extracts of pashanbhed might be due to either desolvation of calcium oxalate crystals or the inhibition of the formation of oxalate. Moreover, the literature shows that the plant extract enriched within phenolic compounds, flavonoids, and isoflavonoids can lead to relaxation of smooth muscle of the urinary and biliary tract which could enable the ejection of stones from the kidney and reduced the size of calculi in rats [18]. The phytochemical analysis of mother extract and DCM fraction confirmed the enrichment of flavonoid, a phenolic compound, and tannins. The ethylene glycol is metabolized to acidic metabolites such as oxalic acid, hippuric acid, benzoic acid, and formic acid which cause metabolic acidosis. The leakage of renal calcium is associated with the gut absorption of calcium and bone calcium release resulting in hypercalcemia and hypercalciuria [6]. In the present study, a similar change was observed; ethylene glycol administration resulted in increase in calcium level in urine, serum, and kidney homogenate thereby promoting the formation of CaO_*x*_ stones. The treatment of mother extract and DCM fraction alone reduced calcium in the urine, serum, and kidney homogenate in urolithiasis rats and thereby prevents the formation and aggregation of stone. The antiurolithiasis activity of* B. ligulata* has been attributed to the high phenolic contents and particularly flavonoids have antiurolithiasis activity [23]. An increase in urinary phosphate is observed in urolithiasis rats. Epitaxially induction of calcium oxalate deposition resulted in stone development by forming calcium oxalate crystals and that was due to the increment of urinary phosphate excretion along with oxalate stress [24]. In our study, it has been observed that there was an increase in calcium and oxalate levels in kidney homogenate in ethylene glycol induced urolithiasis rats. That is to say, ethylene glycol increased the deposition of calcium and oxalate contents in renal tissue and that was significantly reduced by administration of mother extract and DCM fraction alone. Other probable reasons behind the antiurolithiasis potential of mother extract and DCM fraction of* B. ligulata* alone are either it increased bioavailability of nitric oxide which activates cGMP that controls intracellular calcium level [18] or activates various oxalate synthesizing enzymes such as lactate dehydrogenase and glycolic acid oxidase, which catalyze the oxidation and reduction of glyoxalate into glycolate and oxalate [25]. These above changes induce the hyperoxaluria and succeeding CaO_*x*_ crystal adherence and retention in renal tubules [26]. In our present study, raise in serum creatinine, urea, and uric acid had been noted which was ameliorated by treatment with mother extract and DCM fraction of* B. ligulata* alone and thereby improved renal functions in urolithiasis condition.

Histopathological evaluation of kidney through microscopic examination showed accumulation of calcium oxalate deposits inside the tubules in calculi-induced animals. Marked changes such as dilation of the proximal tubules along with interstitial inflammation were observed in urolithiasis rats. The mother extract and DCM fraction alone treated significantly decreased the number and size of calcium oxalate deposits in different parts of renal tubules and also reduced damage to the renal tubules. This may be due to the presence of antioxidant metabolites such as catechin, gallic acid, and quercetin which protect the kidney from oxidative injury by oxalate and crystal deposition [17].

Since the ancient time of human history plants have been used to treat various ailments, for the reason that the herbals have been generally considered to be safe and nontoxic as compared to synthetic medicine. In Indian traditional system of medicine, different parts of plants were being used and their pharmacological properties have also proved [20]. In the human body, the liver performs a number of functions and these are the production of bile, plasma protein synthesis, and detoxification of most substances, whereas spleen also played an important role in removing ruptured, defective blood cells and platelets, platelets storage, and production of blood cells [20]. Histopathological results revealed no serious damage in the hepatic structure and spleen by used concentration. The administration of* B. ligulata* extract having flavonoids can prevent liver tissue injury in a normal pattern. Damaging of liver cells depends on the dose of the extract [20], indicating that our selected dose is appropriate and it can be used for the management of urolithiasis. In summary, it was demonstrated that* B. ligulata* extract does not have any toxic effect at the mentioned dose.

## 5. Conclusion

In summary, the best bioactive fraction (DCM fraction) of mother extract of* B. ligulata* has the curative property against urolithiasis. This study will help further in the discovery of novel phytochemicals from the DCM fraction of mother extract whereas marker analysis will help in metabolic and pharmacokinetic study. However, DCM fraction may be explored as new phytopharmaceuticals drug in future. On the other hand, it may provide a ray of hope in the discovery of new drugs for the statistically neglected area of diseases. In vitro and ex vivo studies will be the new approach for determination of antiurolithiasis potential, which can be used for other drugs as well. The activity may be due to the rich sources of phenolic, polyphenolic, tannins, and flavonoid metabolites in both DCM fraction and mother extracts of pashanbhed. Further studies on metabolomic profiling and pharmacokinetic parameters are still in progress in our laboratory.

## Supplementary Material

Supplementary Figure 1: Sectional view of liver of (a) Normal control, (b) Sham control (c) Urolithiatic control, (d) mother extract, (e) DCM fraction and (f) Neeri tablet treated rats. Supplementary Figure 2: Sectional view of spleen of (a) Normal control, (b) Sham control, (c) Urolithiatic control, (d) mother extract, (e) DCM fraction and (f) Neeri tablet treated rats.

## Figures and Tables

**Table 1:** Percentage of yield of extract and metabolite content in the different extract of *B. ligulata.*

Extract	Yield (% w/w)	Percentage in dried extract (% w/w)			
		Catechin	Gallic acid	Tannic acid	Quercetin
Mother extract	35.9 ± 0.11	2.4 ± 0.22	2.8 ± 0.09	3.1 ± 0.13	4.2 ± 0.18
Hexane fraction	3.4 ± 0.78	0.004 ± 0.09	—	0.3 ± 0.11	—
Toluene fraction	2.9 ± 0.14	0.72 ± 0.11	0.09 ± 0.06	2.8 ± 0.07	—
DCM	5.4 ± 0.26	2.02 ± 0.14	0.89 ± 0.09	6.9 ± 0.14	1.51 ± 0.09
*n*-Butanol	7.5 ± 0.14	2.19 ± 0.08	1.25 ± 0.11	12.1 ± 0.08	1.62 ± 0.11
Water	11.3 ± 0.89	5.34 ± 0.12	7.25 ± 0.12	14.3 ± 0.06	0.78 ± 0.06

Data are expressed as mean ± SEM (*n* = 6).

**Table 2:** Effect of *B. ligulata* on urine parameters of various urolithiasis factors of ethylene glycol induced urolithiasis in Wistar rats.

Group	Urine parameter (mg/24 h)				
	Calcium	Oxalate	Phosphate	Uric acid	Albumin
Normal control	6.1 ± 0.14	1.5 ± 0.11	5.1 ± 0.13	4.7 ± 0.13	0.1 ± 0.09
Urolithiatic control	10.4 ± 0.09^*∗∗∗*^	3.2 ± 0.12^*∗∗∗*^	9.3 ± 0.07^*∗∗∗*^	7.2 ± 0.16^*∗∗∗*^	0.6 ± 0.07^ns*∗*^
Mother extract	7.8 ± 0.14^###^	2.4 ± 0.14^###^	5.8 ± 0.16^###^	5.8 ± 0.21^###^	0.3 ± 0.09^ns#^
DCM fraction	7.4 ± 0.08^###^	2.1 ± 0.17^###^	6.2 ± 0.11^###^	5.2 ± 0.16^###^	0.2 ± 0.08^ns#^
Neeri	7.3 ± 0.25^###^	1.9 ± 0.18^###^	6.2 ± 0.16^###^	5.3 ± 0.09^###^	0.2 ± 0.10^ns#^
Sham (ME)	5.8 ± 0.21^ns*∗*^	2.1 ± 0.15^*∗*^	5.3 ± 0.13^ns*∗*^	5.1 ± 0.11^ns*∗*^	0.1 ± 0.07^ns*∗*^

Data are expressed as mean ± SEM of 6 rats (*n* = 6); ^ns^*p* > 0.05, ^*∗*^*p* < 0.05, and ^*∗∗∗*^*p* < 0.001; *∗* denotes that data were compared with normal control and # denotes that data were with urolithiatic control ^###^*p* < 0.001.

**Table 3:** Effect of mother extract and DCM fraction of *B. ligulata* on various urolithiasis factors in serum parameters of ethylene glycol induced urolithiasis in Wistar rats.

Group	Serum parameter (mg/dL)							
	Calcium	Oxalate	Phosphate	Uric acid	Urea	Creatinine	Albumin	Total protein
Normal control	6.5 ± 0.14	1.2 ± 0.08	4.3 ± 0.13	2.4 ± 0.08	38 ± 1.14	0.7 ± 0.12	4.2 ± 0.21	8.2 ± 0.08
Urolithiatic control	10.3 ± 0.11^*∗∗∗*^	3.3 ± 0.12^*∗*^	7.5 ± 0.06^*∗∗∗*^	4.3 ± 0.09^*∗*^	54 ± 1.12^*∗∗∗*^	1.7 ± 0.14^ns*∗*^	6.4 ± 0.11^*∗∗*^	9.4 ± 0.14^ns*∗*^
Mother extract	7.4 ± 0.16^###^	2.8 ± 0.07^ns#^	4.5 ± 0.24^###^	1.4 ± 0.12^###^	46 ± 2.12^ns#^	0.8 ± 0.17^ns#^	3.8 ± 0.13^###^	8.3 ± 0.13^ns#^
DCM fraction	7.7 ± 0.21^###^	2.6 ± 0.15^ns#^	3.9 ± 0.15^###^	1.8 ± 0.13^##^	39 ± 1.11^ns#^	0.8 ± 0.14^ns#^	4.0 ± 0.18^##^	7.3 ± 0.09^#^
Neeri	8.2 ± 0.09^#^	1.8 ± 0.07^ns#^	2.7 ± 0.06^###^	1.6 ± 0.14^###^	32 ± 0.87^##^	0.8 ± 0.16^ns#^	4.1 ± 0.17^##^	7.7 ± 0.18^ns#^
Sham (ME)	5.4 ± 0.12^ns*∗*^	0.9 ± 0.17^ns*∗*^	4.2 ± 0.11^ns*∗*^	1.9 ± 0.09^ns*∗*^	42 ± 0.78^ns*∗*^	0.7 ± 0.21^ns*∗*^	3.9 ± 0.27^ns*∗*^	7.6 ± 0.19^ns*∗*^

All values are expressed as mean ± SEM of 6 rats (*n* = 6); ^ns^*p* > 0.05, ^*∗*^*p* < 0.05, ^*∗∗*^*p* < 0.01, and  ^*∗∗∗*^*p* < 0.001; *∗* denotes that data were compared with normal control and # denotes that data were with urolithiatic control ^###^*p* < 0.001. ^##^*p* < 0.01.

**Table 4:** Effect of *B. ligulata* on various urolithiasis factors in kidney homogenate of ethylene glycol induced urolithiasis in Wistar rats.

Group	Kidney homogenate parameter (mg/100 mg kidney weight)			
	Calcium	Oxalate	Phosphate	Uric acid
Normal control	3.3 ± 0.11	1.6 ± 0.09	2.5 ± 0.11	2.1 ± 0.11
Urolithiatic control	5.7 ± 0.12^*∗∗∗*^	2.3 ± 0.08^*∗∗*^	3.8 ± 0.08^*∗∗∗*^	3.5 ± 0.09^*∗∗∗*^
Mother extract	4.1 ± 0.13^###^	1.8 ± 0.07^#^	2.8 ± 0.11^##^	2.6 ± 0.08^##^
DCM fraction	3.8 ± 0.12^###^	1.7 ± 0.06^##^	2.4 ± 0.04^###^	2.4 ± 0.07^###^
Neeri	3.5 ± 0.14^###^	1.5 ± 0.08^###^	2.2 ± 0.07^##^	2.3 ± 0.06^###^

Data are expressed as mean ± SEM of 6 rats (*n* = 6); ^ns^*p* > 0.05, ^*∗*^*p* < 0.05, ^*∗∗*^*p* < 0.01, and ^*∗∗∗*^*p* < 0.001; *∗* denotes that data were compared with normal control and # denotes that data were with urolithiatic control ^###^*p* < 0.001. ^##^*p* < 0.01.
