# Supplementary material for: Antiurolithiasis Activity of Bioactivity Guided Fraction of Bergenia ligulata against Ethylene Glycol Induced Renal Calculi in Rat
Source: Biomed Res Int. 2017 Mar 2;2017:1969525. doi: 10.1155/2017/1969525 (PMC5352974; doi:10.1155/2017/1969525)

**Supplementary Figure 1:** Sectional Supplementary Figure 1 showed the sectional view of liver of (a) Normal control, (b) Sham control (c) Urolithiatic control, (d) mother extract, (e) DCM fraction and (f) Neeri tablet treated rats.

**
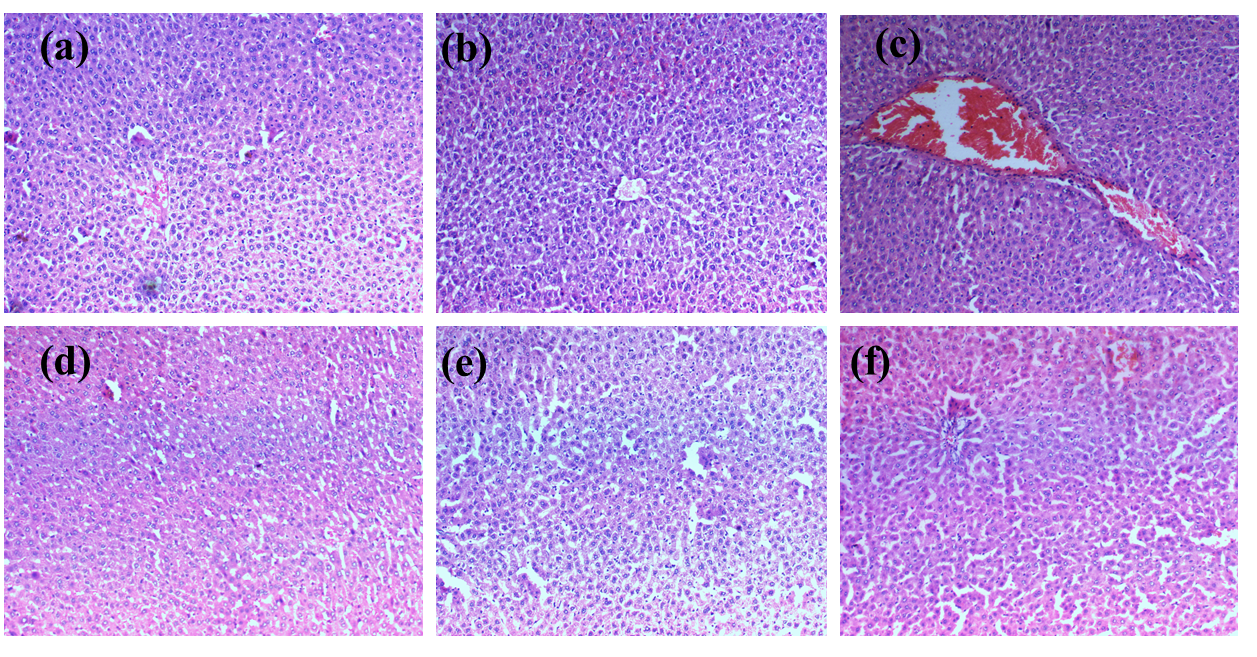
**

**Supplementary Figure 2**: Sectional view of spleen of (a) Normal control, (b) Sham control, (c) Urolithiatic control, (d) mother extract, (e) DCM fraction and (f) Neeri tablet treated rats.


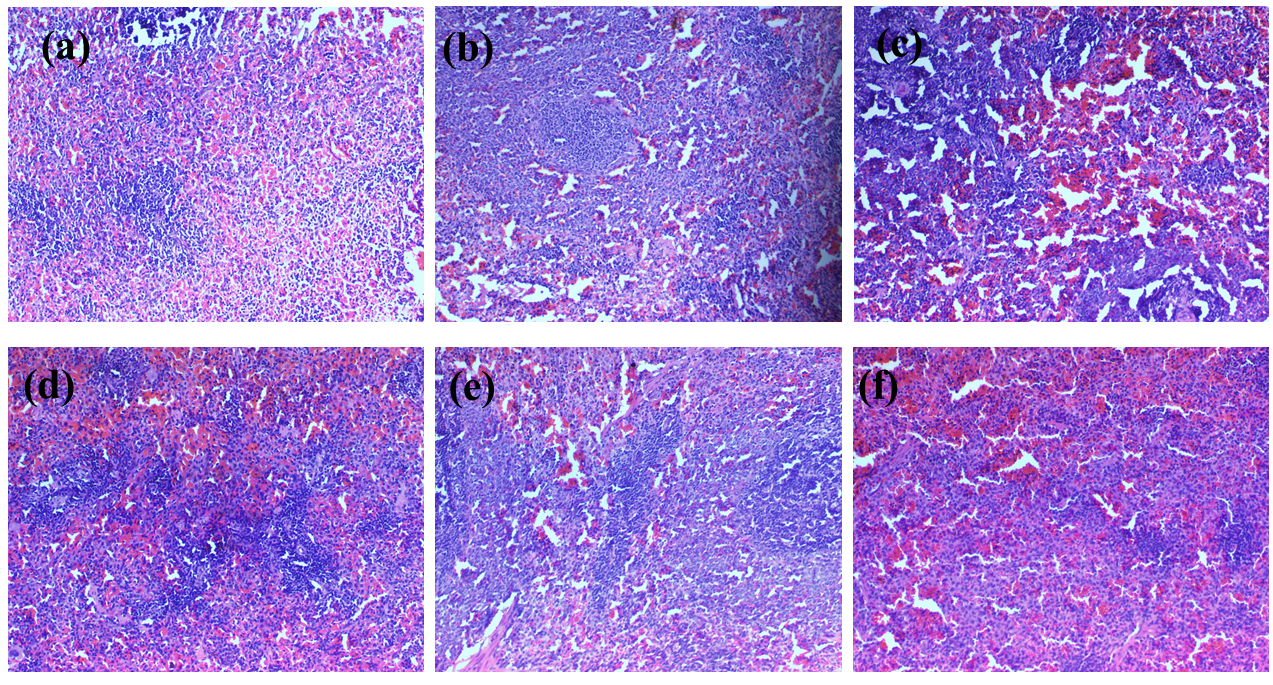

Supplement: Supplementary file 1 — Supplementary Figure 1: Sectional view of liver of (a) Normal control, (b) Sham control (c) Urolithiatic control, (d) mother extract, (e) DCM fraction and (f) Neeri tablet treated rats. Supplementary Figure 2: Sectional view of spleen of (a) Normal control, (b) Sham control, (c) Urolithiatic control, (d) mother extract, (e) DCM fraction and (f) Neeri tablet treated rats. [file 1969525.f1.docx]
